# From virus isolation to metagenome generation for investigating viral diversity in deep-sea sediments

**DOI:** 10.1038/s41598-017-08783-4

**Published:** 2017-08-21

**Authors:** Cinzia Corinaldesi, Michael Tangherlini, Antonio Dell’Anno

**Affiliations:** 1 Department of Sciences and Engineering of Materials, Environment and Urbanistics, Polytechnic University of Marche, Via Brecce Bianche, 60131 Ancona, Italy; 2 Department of Environmental and Life Sciences, Polytechnic University of Marche, Via Brecce Bianche, 60131 Ancona, Italy

## Abstract

Viruses are the most abundant and, likely, one of the most diverse biological components in the oceans. By infecting their hosts, they play key roles in biogeochemical cycles and ecosystem functioning at a global scale. The ocean interior hosts most of the microbial life, and, despite deep-sea sediments represent the main repository of this component and the largest biome on Earth, viral diversity in these ecosystems remains almost completely unknown. We compared a physical-chemical procedure and a previously published sediment washing-based procedure for isolating viruses from benthic deep-sea ecosystems to generate viromes through high-throughput sequencing. The procedure based on a physical-chemical dislodgment of viral particles from the sediments, followed by vacuum filtration was much more efficient allowing us to recover >85% of the extractable viruses. By using this procedure, a high fraction of viral DNA was recovered and new viromes from different benthic deep-sea sites were generated. Such viromes were diversified in terms of both viral families and putative functions. Overall, the results presented here provide new insights for evaluating benthic deep-sea viral diversity through metagenomic analyses, and reveal that deep-sea sediments are a hot spot of novel viral genotypes and functions.

## Introduction

Viruses are key agents of prokaryotic mortality in the global oceans and by killing their hosts they play an important role in the functioning of marine food webs and carbon and nutrient cycling^1, 2^. Previous studies revealed that viruses have a major role in benthic deep-sea ecosystem functioning^3, 4^, but their diversity is still largely unknown.

Available information suggests that thousands to more than a million viral genotypes can be present in marine ecosystems^5, 6^ although recent studies carried out in open ocean systems suggest that viral diversity could be lower than expected^7, 8^. Exploring viral diversity in environmental samples is challenging due to the absence of conserved genes in the viral genomes (e.g., the ribosomal genes of the prokaryotes and eukaryotes)^9^, and because most of the viral hosts cannot be cultivated^5, 10^.

The advent of metagenomic approaches has represented a novel opportunity for characterizing viral diversity as it allows us to capture, to a large extent, the genetic richness of viral assemblages in marine ecosystems^5–9, 11^. While a number of studies have investigated viral diversity in the water column^8, 12, 13^, only two investigations, one in shallow sediments and the other in deep-sea floor, have been carried out so far in benthic ecosystems^14, 15^. Since the viral richness reported for the sediments appeared to be much higher than that of the adjacent water column, it was suggested that benthic ecosystems can represent one of the largest reservoir of viral diversity on the planet^14^.

The assessment of viral diversity through metagenomic analyses involves four main steps: i) the recovery and concentration of viral particles, ii) the extraction and purification of viral DNA, iii) the high-throughput sequencing of viral DNA, and iv) bioinformatic analyses^13, 16, 17^. Different protocols have been used to recover viruses from water samples including ultracentrifugation, chemical flocculation, size fractionation and tangential flow filtration^12, 13, 18, 19^. Such protocols cannot be applied directly to sediment samples since viral particles need to be dislodged and recovered from the sedimentary matrix before purification from non-viral particles (i.e. prokaryotes and micro-eukaryotes)^19^.

To date, the approach utilized to generate viral metagenomes from sediments, as well as other solid matrices (e.g., stromatolites), is based on washing steps with saline buffers for isolating viral particles without taking into account their extraction efficiency^14, 15, 19, 20^. However, the development of a procedure able to recover the highest number of viruses present in the sediments is essential to generate viral metagenomes that can better reflect the actual composition of benthic viral assemblages. Specific protocols for the analysis of viral abundance in different typologies of marine sediments have been previously developed^21, 22^. However, these protocols are not appropriate to investigate viral diversity through metagenomics. Generating viromes from the sediments also requires specific steps for their concentration and purification from non-viral particles. In this regard, one of the most used techniques for viral concentration and purification is based on the density gradient ultracentrifugation^19^, although it is time-consuming, requires specific instrumentations and can result in a significant viral loss^19, 23^. Moreover, recent studies revealed that viral particle purification, based exclusively on a DNase treatment, can provide similar results in term of viral diversity when compared with more sophisticated procedures such as the density gradient ultracentrifugation^24, 25^.

Here, we present a procedure for generating viral metagenomes from surface deep-sea sediments, which combines a physical-chemical treatment and vacuum filtration for the isolation, purification and concentration of benthic viruses. The reliability of this procedure was assessed through a comparison with protocols previously applied to marine sediments^14, 15^ and other marine matrices^19, 20^. This study allowed us to generate new viromes, thus representing a starting point for expanding our knowledge on viral diversity in benthic deep-sea ecosystems.

## Results

### Recovery of viral particles from sediments

The viral abundances determined following the physical-chemical procedure (PC procedure) applied to the sediment samples from the NE Atlantic sites 1 and 2 and the Mediterranean Sea ranged from 3.65 ± 0.31 × 10^8^ to 6.86 ± 1.14 × 10^8^ viruses per gram of dry sediment (Table 1). The viral abundances determined by using a sediment washing-based procedure (WB procedure) were ca. 100-200-times lower (2.39 ± 1.20 × 10^6^ and 3.74 ± 1.37 × 10^6^ viruses per gram of dry sediment in the NE Atlantic 1 and Mediterranean Sea sites, respectively; Table 1). The total viral abundances determined by using the procedure developed exclusively for viral counting on diluted sediments without the centrifugation step (see method section) were 28–34% higher than those determined in the same sites following the PC procedure (Table 1).

**Table 1:** Comparison among the abundances of viruses extracted from sediments by means of the WB and PC procedures (before the step for concentrating viruses using vacuum filtration or TFF treatment) and the procedure developed for viral counting^21^. All data are expressed as 10^8^ virus g^−1^. n.a = not available.

	PC procedure	WB procedure	Procedure for viral counting
NE Atlantic 1	4.95 ± 0.643	0.0239 ± 0.012	6.86 ± 0.685
NE Atlantic 2	6.86 ± 1.14	n.a.	9.59 ± 0.923
Mediterranean	3.65 ± 0.305	0.0374 ± 0.0137	4.75 ± 0.397

All data are expressed as 10^8^ virus g^−1^. n.a = not available.

The analysis of the viral abundance obtained after tangential flow filtration (TFF) indicated a loss of viruses higher than 80% when compared with the abundance of viruses recovered from sediments by means of the PC procedure before this concentration step (Table 2). Such a loss was significantly lower when the vacuum filtration procedure was directly used for concentrating viruses (10–15%; ANOVA, p < 0.01; Table 2).

**Table 2:** Comparison among the abundances of viruses extracted from sediments by means of the PC procedure before and after vacuum filtration and TFF treatment.

	Before step for concentrating viruses	After vacuum filtration	After TFF treatment
NE Atlantic 1	4.95 ± 0.643	3.59 ± 0.66	0.866 ± 0.182
NE Atlantic 2	6.86 ± 1.14	6.36 ± 1.14	1.05 ± 0.115

All data are expressed as 10^8^ virus g^−1^.

### Viral DNA extraction efficiency

**Figure 1:**
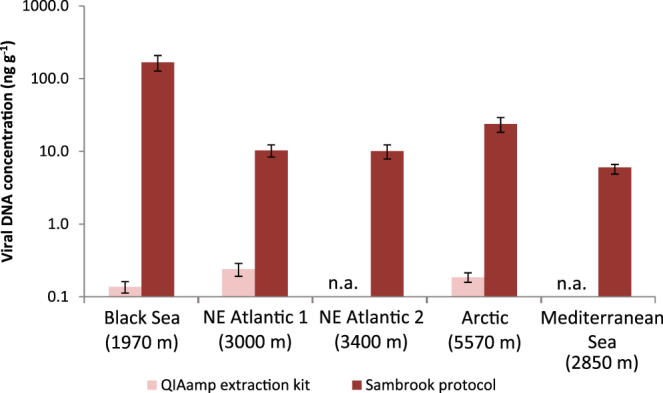
Comparison of viral DNA concentrations obtained using the QIAamp DNA extraction kit and the Sambrook protocol. Mean (n = 3) and standard deviations are shown. n.a. = not available.

**Figure 2:**
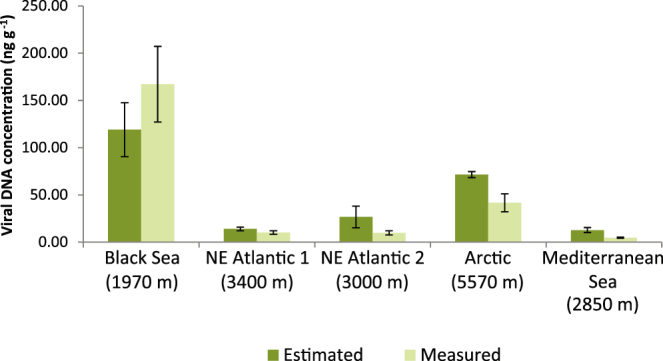
Comparison between viral DNA concentrations measured fluorometrically and DNA concentrations estimated by converting viral abundances into DNA equivalents. Mean (n = 3) and standard deviations are shown.

The Sambrook protocol for viral DNA extraction, applied on viruses concentrated by vacuum filtration, always provided significantly higher viral DNA concentrations (4.7 ng DNA g^−1^ of sediment at the Mediterranean Sea site, and 167 ng DNA g^−1^ of sediment at the Black Sea site) when compared to those obtained by using the QIAamp kit (Fig. 1). The Sambrook protocol applied to samples processed with the PC procedure provided viral DNA concentrations similar to those obtained by using the protocol reported by Thurber *et al*.^19^ (106 ± 26 and 12 ± 3 ng DNA g^−1^ of sediment, at the Black Sea and Arctic sites, respectively). The quantity of viral DNA extracted from the sediments through the Sambrook protocol accounted, on average, for ca. 60% of the amount of DNA obtained converting the viral abundances measured following the PC procedure by epifluorescence microscopy into DNA equivalents (Fig. 2).

The concentration of viral DNA extracted by using the Sambrook protocol from the samples processed with the WB procedure and vacuum filtration was <2 pg DNA g^−1^ dry sediment. Therefore, viral DNA obtained by the combination of PC, vacuum filtration and Sambrook/Thurber procedures can better reflect the composition of viral assemblages in deep-sea sediments.

qPCR analyses used to check for the potential contamination by non-viral DNA revealed that the copy number of 18S and 16S rRNA genes was below the detection limit in all samples (i.e. below 25–30 ribosomal gene copies per g of sediment).

### Quality check of viral metagenomes

Number of reads pre-quality check (Pre-QC), post-quality check (Post-QC) and after contamination removal, mean length of the reads, mean GC content, total number of sequenced bases obtained by pyrosequencing of viral metagenomes generated by using the PC procedure and MDA treatment are reported in the Supplementary Information (Table S1).

After pyrosequencing, the total number of sequences passing all the quality control steps (including contamination removal) ranged from ca. 65,000 for the Mediterranean Sea site to more than 165,000 for the NE Atlantic site 2. The average read length varied from 484 to 546 bp and GC content from 42 to 49%. Contaminating sequences accounted for 0.03%, 0.1% and 7.7% of all reads removed from the viromes (including sequences after quality check and de-replication) from the NE Atlantic site 1, Arctic and Mediterranean samples, respectively, and were exclusively due to the presence of human genomic sequences. No contaminating sequences were detected in the Black Sea and NE Atlantic site 2 (Table S1).

After contamination removal, the additional analyses carried out by hmm_rRNA indicated a negligible eukaryotic and prokaryotic ribosomal DNA contamination in the viromes generated (0 to 11 reads affiliated to rRNA genes).

Finally, the analysis of dinucleotide frequencies showed that viromes generated in the present study were more similar to other viromes from different environmental matrices than to previously published microbial metagenomes (Figure S1).

### Taxonomic affiliation of viromes

**Figure 3:**
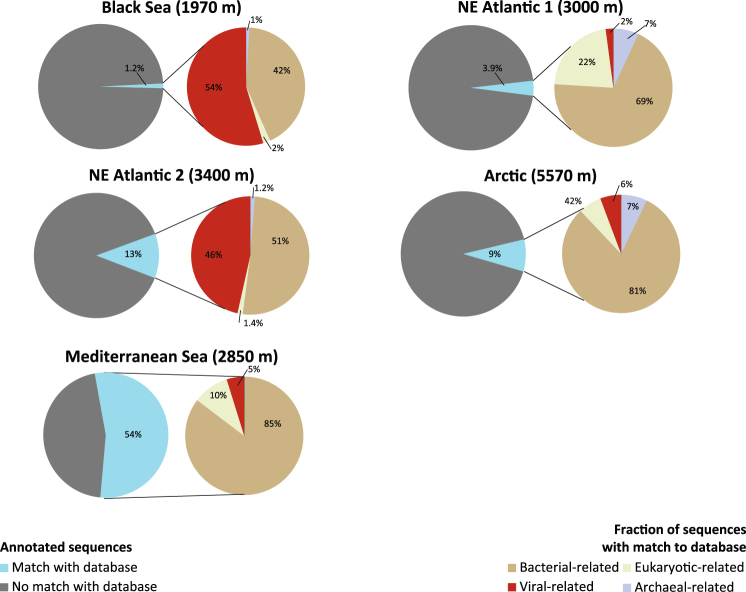
The pie charts on the left show the contributions of the identified sequences to the total sequences obtained from the pyrosequencing analysis of the five viromes (MG-RAST, RefSeq database, 10^−5^ E-value cutoff). The cross sections of the pie charts on the right show the contributions of the sequences associated with viruses and to the different domains of life.

Reads with a hit to the RefSeq database in the MG-RAST system ranged from ca. 1% to 54% of the total number of reads (at the Black Sea and Mediterranean sites, respectively; Fig. 3). Within the annotated fraction of reads, the contribution of viral-associated sequences ranged from 2% to 54% (at the NE Atlantic site 1 and Black Sea site, respectively; Fig. 3). At the Black Sea site and NE Atlantic site 2 the majority of the annotated reads was represented by viral-associated sequences (54% and 47%, respectively), whereas at the Mediterranean and Arctic sites bacterial-associated sequences dominated (85% and 80%, respectively). The highest fraction of eukaryotic-associated sequences was found at the NE Atlantic site 1 (22%), whereas the lowest at the NE Atlantic site 2 (ca. 1%). Archaeal-associated sequences contributed for up to 7% in the Arctic site.

**Figure 4:**
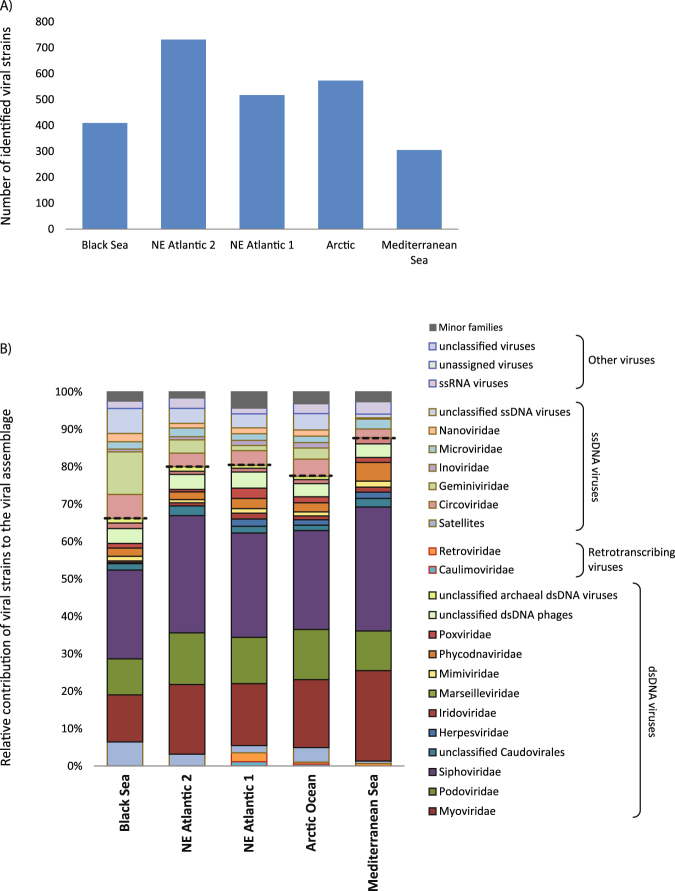
Viral genotype richness (**A**) and taxonomic composition of the viral assemblages (**B**) for each site investigated. The taxonomic composition is expressed as the contribution of viral genotypes to the different viral families. Dashed lines were used to separate ssDNA and dsDNA viral families.

The number of viral genotypes identified in our metagenomes ranged from 305 to 731 in the Mediterranean and NE Atlantic site 2, respectively (Fig. 4a). The viral assemblage composition (defined as the contribution of viral genotypes to the different families) was dominated by dsDNA viruses in all viromes generated (up to 77% in the Mediterranean site; Fig. 4b). In all deep-sea viromes, viral genotypes identified belonged mostly to the order Caudovirales (48–66%). The most represented families, within the order Caudovirales, were *Siphoviridae* followed by *Myoviridae* and *Podoviridae*. The Nonpareil diversity indices for all samples ranged from 13.4 (for the Black Sea sample) to 16.3 (for the NE Atlantic 2 sample; Figure S2).

The results of the cluster analysis (based on Bray-Curtis similarity) applied to the viral taxonomic composition in terms of viral genotypes revealed a low similarity among the viromes generated from the different benthic deep-sea sites (Figure S3a). This was also confirmed by the output of the cluster analysis carried out using tetranucleotide frequencies (Figure S3b), which highlighted an average distance up to >0.8.

The distance-based redundancy analysis (dbRDA), used to assess the relationships between the composition of the viral assemblages and environmental characteristics of the different benthic deep-sea sites, showed that the first dbRDA axis captured 63.8% of the variance of the viral assemblage composition whereas the second dbRDA axis captured 23.4% of the variance (Figure S4). The composition of viral assemblages at each site showed relationships with different environmental factors. In particular, the viral assemblage composition was mainly related with organic C load in the sediments of the Black Sea site (r = −0.714), with temperature (r = 0.732) in the Mediterranean site and with salinity (r = 0.395) in the Arctic Ocean site.

Rarefaction curve analyses of sequence clusters showed that the virome of the NE Atlantic site 2 contained the highest number of sequence clusters, whereas the virome of the Mediterranean site was characterized by the lowest value (Figure S5a). This pattern was maintained even when a sub-sampling strategy (by using 50000 sequences sampled randomly) was applied (Figure S5b).

**Figure 5:**
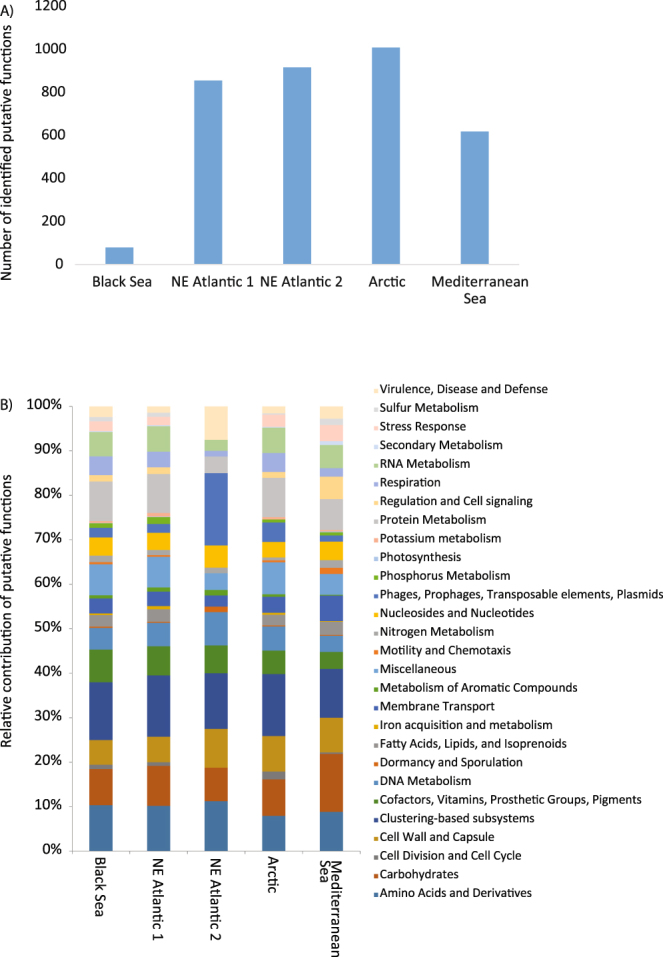
Putative functional richness (**A**) and contribution of functions belonging to each functional class to the total number of functions (**B**) identified by MG-RAST and Subsystems database (with default parameters) within each virome.

The highest number of putative functions was observed in the virome from the Arctic site whereas the lowest number in the virome from the Black Sea (Fig. 5a). Similar functional classes were represented in our viromes (Fig. 5b), but the contribution of putative viral functions to the different functional classes was very different (similarity <50%; Figure S6).

### Viral assemblage composition in MDA treated and untreated samples

**Figure 6:**
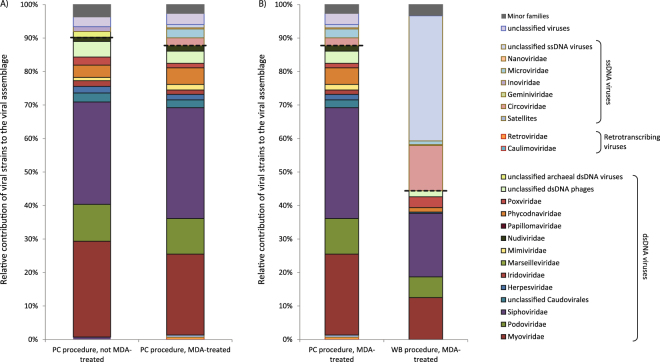
Comparison between the taxonomic composition of viromes of the Mediterranean Sea site generated in MDA-treated and un-treated samples (**A**) and by using WB and PC procedures (**B**). The taxonomic composition is expressed as the contribution of viral genotypes to the different viral families. Dashed lines were used to separate ssDNA and dsDNA viral families.

The contribution of known ssDNA viral genotypes in the un-treated virome was lower than in the MDA-treated sample (1% vs. 6%, respectively; Fig. 6A). Despite the untreated sample contained a higher number of dsDNA viral genotypes than the MDA-treated sample (578 vs. 305), the composition of the dsDNA viral assemblages in the two viromes was quite similar (Fig. 6A). Indeed, viral genotypes affiliating with the order Caudovirales (including the families *Myoviridae, Podoviridae, Siphoviridae*) in the MDA-treated and untreated viromes showed almost equal contributions (70% and 71%, respectively). Also the contributions of other less relevant viral genotypes (e.g., *Phycodnaviridae*, *Mimiviridae)* were quite similar.

### Comparison between viromes generated with the PC procedure and the WB procedure

The virome generated using the PC procedure was characterized by a much higher number of viral genotypes (305) than using the WB procedure (65). Some viral families (i.e., *Herpesviridae*, *Iridoviridae* and *Phycodnaviridae*) were found exclusively in the virome generated using the PC procedure. Moreover, the relevance of dsDNA viral genotypes in the sample generated with the PC procedure was higher than using the WB procedure (89% *vs*. 70% respectively; Fig. 6B).

## Discussion

Viral diversity in benthic deep-sea ecosystems is still largely unknown. The remoteness and the consequent technological difficulties for investigating deep-sea ecosystems^26^ and the methodological difficulties in isolating viral genomes has certainly contributed to make this kind of investigations very challenging^15^.

In the present study, we found that the abundances of viruses recovered using the PC procedure were up to more than two order of magnitude higher than those obtained with the WP procedure.

These results indicate that the PC procedure is the most efficient method available to date for maximizing the recovery of viruses suitable for the analysis of their diversity in surface deep-sea sediments. Despite the PC procedure maximizes the extraction of viruses from sediments, the centrifugation step included in this method determined the loss of ca. 30% of the extractable viruses from sediment samples. However, this step is essential to reduce the background due to sediment particles, which especially in deep-sea samples hamper the recovery of viruses and their DNA for further metagenomic analyses.

The PC procedure is a non-labor-intensive method that can be performed on small volumes of sediment and can be potentially applied also to other complex and solid matrices (e.g. soils and microbial mats). Since the comparison between the PC and WP procedures has been carried out on surface sediments, further studies are needed to test for the efficiency of PC procedure on the extraction of viruses present in deeper sediment layers.

The pre-filtration step onto 0.2 µm filters used in this procedure is commonly carried out to avoid contamination due to the presence of cellular organisms^12, 13, 15, 27, 28^, although it can result in the selective exclusion of a certain fraction of viruses larger than 0.2 µm and/or absorbed to suspended inorganic particles. However, in the present study no significant loss of viruses due to filtration through 0.2 µm pore size filters was observed (i.e. similar viral counts before and after filtration step; Table 2). This is likely due to the fact that: 1) most of the viruses have a size smaller than 0.2 µm, as previously highlighted by Transmission Electron Microscopy^3, 29^ and metagenomic analyses^30^; 2) a large amount of the sediment particles, which could adsorb viruses, were already removed by centrifugation (thus allowing the majority of viruses to pass through the pores of 0.2 µm filters), and 3) the retention of viruses due to the progressive clogging of the 0.2 µm filters was minimized since several filters during the filtration step were used.

Another critical point to generate viral metagenomes is the concentration of viruses after their recovery from the sediments. Despite tangential flow filtration being one of the most utilized method to concentrate viruses from environmental samples^27, 28, 30, 31^, we found that such a procedure resulted in a significant loss of viral particles (on average ca. 80%), very close to that previously reported for seawater samples^23^. Other studies, carried out on aquatic samples, reported that TFF caused a significant loss of viruses when compared with polyethylene glycol (PEG) precipitation and ultracentrifugation^32^. Although such approaches were not included in our comparative analysis, our findings revealed that concentrating directly viral particles by using vacuum filtration allowed us to recover 85–90% of extractable viral particles (i.e. obtained after the first extraction plus three further washing steps). Moreover, vacuum filtration combined with a DNase treatment allows us to avoid the purification of viruses from non-viral particles through density gradient ultracentrifugation, which has been reported to be not only time-consuming and expensive, but can also have different limitations (e.g., virions above or below predicted densities, damaged virions)^19, 33^.

The extraction efficiency of viral DNA depends on the procedure used^19^. We found that the viral DNA concentrations obtained by using the QIAamp DNA extraction kit were much lower than those obtained by using traditional laboratory procedures (Sambrook’s procedure and Thurber’s protocol)^19, 33^ based on physical-chemical lysis of viral capsids. This is consistent with previous findings that revealed that the extraction of total DNA pools from deep-sea sediments by means of commercial extraction kits was significantly lower than by using a classical laboratory protocol^34^.

Overall, the preparation of DNA from benthic viruses using the PC procedure combined with vacuum filtration and Sambrook protocol was efficient, accounting for ca. 60% of the amount of DNA obtained by converting the abundances of extractable viruses into DNA equivalents using a viral genome content of 0.039 fg DNA per virus^35^. Such a contribution would be even higher considering the presence in our metagenomes of ssDNA viruses, whose DNA content is lower than in dsDNA viruses (viralzone.expasy.org/all_by_species/239.html).

Our results also indicate that, excluding the Black Sea site (which does not reflect the typical environmental characteristics of benthic deep-sea ecosystems due to anoxic and eutrophic conditions), the amounts of viral DNA extracted from 50 g of wet sediment using the PC procedure were not sufficient for a direct sequencing analysis. By using this procedure, we have estimated that ca. 60–130 g of wet deep-sea sediment would be required for direct high-throughput sequencing. Such an amount of sediment required for direct sequencing would be much higher if the WB procedure is used since the recovery of viral DNA even using 500 g of wet sediment was very low (<1 ng of DNA).

Multiple displacement amplification (MDA), which is generally used to increase the amounts of viral DNA to perform metagenomic analyses^8, 15, 30, 36^, was used on viral DNA recovered by using both the PC and WB procedures. We found that viral genotype richness was much higher in the virome generated with the PC procedure than in the virome generated with the WB procedure and that some viral families were present exclusively in the virome generated with the PC procedure. These findings indicate that the use of the WB procedure can lead to a major underestimation of the viral diversity in deep-sea sediments. Therefore, the efficiency of viral DNA recovery can influence viral genotype richness and assemblage composition.

The use of MDA, along with the amplification and linker or adaptor ligation steps used for metagenomic library construction, may introduce biases. In this study, we compared viromes generated with and without MDA treatment and we found that, as previously reported, MDA treatment resulted in an over-representation of sequences of ssDNA viruses^37^. Therefore, quantitative estimates of viral genotype richness based on the number of viral reads should be viewed with caution^25, 38–42^. To bypass biases introduced by MDA, we assessed the taxonomic composition of viral assemblages considering the number of viral genotypes belonging to the different viral families and not the number of viral sequences, which could be randomly amplified leading to a mis-representation of the composition of viral assemblages. By using this approach, we provide evidence that, despite the untreated sample displayed a higher number of dsDNA viral genotypes than the MDA-treated sample and *vice-versa* for ssDNA viral genotypes, the composition of the dsDNA viral assemblages in the two viromes was similar.

In the light of technological advancements in sequencing of low DNA quantities (e.g., few nanograms for Illumina), our procedure could allow us to sequence viral DNA even without any pre-amplification step (e.g., MDA and linker amplification). In addition, single-molecule sequencing (e.g., PacBio RS SMRT system) represents a promising approach with limited biases due to library preparation and amplification, but has yet to be optimized for metagenomics^43^. Despite MDA may introduce biases into the analysis of viral assemblage composition, our results indicate that this treatment provides a wider view of the diversity of ssDNA viruses in benthic deep-sea ecosystems, which otherwise can be largely underestimated^44^. Thus, the integration of data from MDA-treated and un-treated viromes obtained from the same sediment sample could improve our knowledge of the actual viral genotype diversity (including both ssDNA and dsDNA viruses) in benthic deep-sea ecosystems.

Despite qPCR analyses carried out before sequencing revealed that the copy number of 18 S and 16 S rRNA genes was negligible, bioinformatic analyses after sequencing indicated the presence of contaminating sequences in three out of five viromes. Such a contamination, although accounting for a very small fraction of the viromes (up to 4% in the Mediterranean virome), was exclusively ascribed to human genomes. We can hypothesize that the stochastic presence of human genomic sequences in some of the viromes generated in the present study could have been introduced during sequencing as already reported in previous investigations^45^.

After contamination removal, the viromes generated using the PC procedure were characterized by a different contribution of non-viral (i.e. cellular organisms-associated especially represented by prokaryotic sequences) sequences. However, as indicated by hmm_rRNA, the number of 16 S and 18 S rRNA genes in the viromes investigated was negligible. Moreover, dinucleotide frequencies analyses revealed that our viromes were more similar to other viromes generated from different environmental matrices than to previously published microbial metagenomes. The presence of cellular organisms-associated sequences, indeed, has been repeatedly documented in other viromes obtained from marine systems^5, 12, 13, 46^. We hypothesize that the presence of cellular organism-associated sequences could be due to gene transfer processes between viruses and their hosts^24, 47^. Therefore, we can conclude that our procedure is selective in the recovery of the large majority of “genuine” viral DNA from deep-sea sediment samples.

We found that the fraction of reads annotated in our viromes was highly variable, indicating that a wide portion of viral sequences remains still unknown and could belong to novel viral genotypes. Also the viral genotype richness was variable among the investigated viromes and this was confirmed by diversity indices calculated by means of the Nonpareil program. All viromes were dominated by dsDNA viruses mainly belonging to *Siphoviridae, Myoviridae* and *Podoviridae* (order Caudovirales), although the contribution of single genotypes to each viral family greatly varied among samples as revealed by the cluster analysis. Such differences in the composition of the whole virome (considering both annotated and not annotated sequences) were also observed by using tetranucleotide frequency analysis. Besides viral DNA, our viromes showed the presence of a limited number of genotypes affiliated with RNA viruses. The presence of such viruses could be due to either: i) the concomitant recovery of a fraction of viral RNA during extraction and purification of DNA, since no digestion step with RNase was applied; or ii) the presence of sequences which are erroneously affiliated with RNA viruses due to the lack of related, yet-unknown DNA viruses available in databases.

Since the same procedure for virome generation was used for all samples, the differences in viral genotype richness and taxonomic composition can be dependent on the different environmental conditions of the biogeographic regions investigated able to influence host diversity^48^. In this regard, the taxonomic composition of the virome from the Black Sea site was mainly related to the organic carbon content in the sediments, whereas that of the Arctic and Mediterranean sites were related to thermohaline conditions.

We also found that the putative functional richness (considering the total number of putative functions and not the number of sequences with a functional annotation) in our viromes was highly variable and uncoupled with the viral genotype richness. Despite a similar contribution of functional classes was found in all viromes, the contributions of putative viral functions to the different functional classes were very different. These results suggest that benthic deep-sea ecosystems characterized by different ecological settings harbor taxonomically diverse viral genotypes with distinct sets of functions which can confer advantages on viruses for interacting with their hosts.

Concluding, results obtained here indicate that our procedure, allowing us to recover most of the viruses contained in the sediments and their genomes, is a powerful tool for a better assessment of the composition of viral assemblages in the most remote and unexplored biome of Earth, thus representing the starting point for improving our understanding of the global benthic viral diversity and their ecological and evolutionary implications.

## Methods

### Sample collection

Undisturbed sediment samples were collected by using a multiple corer in five deep-sea regions. Three independent replicates (n = 3) were collected from each site. One sampling site was located in the Black Sea at 1970 m depth (42°59’54.204″ N, 31°30’58.644″ E), two sites in the NE Atlantic Ocean along the Portuguese Margin at 3530 m and 3060 m depth, respectively (39°30’24.18″ N, −9°50’0.604″ E and 41°43’51.2394″ N, −10°40’56.568″ E, hereafter defined NE Atlantic 1 and NE Atlantic 2 sites), one site in the Arctic Ocean at 5571 m depth (79°8’0.5994″ N, 2°50'32.2794″ E) and one in the Mediterranean Sea at 2850 m depth (39°31'04.1880″ N, 6°10'32.4012″ E). Further details on sampling sites and environmental characteristics are reported in the Supplementary Information.

### Recovery of viral particles from sediments for the analysis of viral diversity

We used a physical-chemical treatment (PC procedure) to dislodge viruses from the sediments. Fifty grams of the deep-sea sediment samples collected in the NE Atlantic Ocean (sites 1 and 2) and from the Mediterranean Sea were diluted with 50 ml of autoclaved virus-free seawater (collected at the water-sediment interface and pre-filtered through 0.02-μm-pore-size filters; Whatman Anodisc) and homogenized at 300 rpm by using a magnetic stirrer (VELP Scientifica, model ARE) for 10 minutes at room temperature. The amount of sediment here utilized corresponds to the quantity of the top 1 cm of sediment recovered from a single core through a multiple corer deployment of the same sediment samples.

We found that the partitioning of the sediment slurry (sediment:seawater, 1:1) in aliquots increased the efficiency of recovery of viruses up to 10-fold when compared with an equal amount of undivided sediment sample. Therefore, the slurry was divided into aliquots of 2 ml in 50 sterile tubes and each aliquot was added with 8 ml virus-free seawater (10 ml final volume) containing tetrasodium pyrophosphate (5 mM final concentration). Samples were incubated for 15 minutes in the dark and then treated by ultrasounds for 3 times of 1 minute each, with 30 seconds of manual shaking after each cycle (frequency: 40 KHz; Bransonic Branson 3510). The samples were centrifuged at 800 × *g* for 10 minutes to reduce the interference due to suspended particles, and the supernatants were collected. The sediment was homogenized again with 4 ml virus-free seawater and centrifuged (800 × *g* for 10 minutes). This step was repeated two times more and the supernatants were combined (final volume ca. 900 ml). Supernatants were incubated with DNase I (2 U/ml) in the dark at room temperature for 15 minutes to remove extracellular DNA (i.e. not associated with cells and/or intact viral particles)^49^.

### Assessing the recovery efficiency of viral particles

To evaluate the recovery efficiency of viruses from the sediments obtained by using the PC procedure, aliquots of the supernatants (i.e. obtained after the centrifugation step) were filtered onto 0.02 µm pore size filters (Anodisc Al_2_O_3_, 25 mm diameter) and the filters were analysed by epifluorescence microscopy (magnification, ×1000)^21^ after staining with SYBR Gold (dilution 1:5000)^50^.

The recovery efficiency of viral particles using the PC procedure was compared with that obtained following a washing-based procedure (defined WB procedure) previously applied to shallow-coastal^14^ and to deep-sea sediment samples with slight modifications^15^. To do this, about 500 g of sediment from the NE Atlantic 1 and Mediterranean Sea sites used for the recovery of viral particles by using the PC procedure, were diluted with 500 ml of autoclaved and 0.02 µm pre-filtered seawater within a previously autoclaved beaker. The slurry was shaken at 300 rpm by using a magnetic stirrer (VELP Scientifica, model ARE) for 10 minutes at room temperature. Then, the slurry was centrifuged at 800 × *g* for 10 minutes to remove suspended particles. After centrifugation the supernatant was recovered and sediments were added again with autoclaved and 0.02 µm pre-filtered seawater and centrifuged at 800 × *g* for 10 minutes. These steps were repeated four times more and all supernatants were combined (ca. 3 L). Aliquots of supernatants were filtered onto 0.02 µm pore size filters (Anodisc Al_2_O_3_, 25 mm diameter) and the filters were analysed by epifluorescence microscopy (magnification, ×1000)^21^ after staining with SYBR Gold (dilution 1:5000)^50^.

The viral abundances obtained by the PC and WB procedures were compared to those determined in additional sediment samples collected from the same sites according to protocols developed for viral counting^21, 22^. For viral counting, sediment samples were first treated with pyrophosphate (final concentration, 5 mM) and ultrasound (three 1-min treatments using a Branson Sonifier 2200; 60 W) to detach viruses adsorbed onto sediment particles. Then, samples were diluted 250-fold with sterile and virus-free water (filtered through 0.02-µm-pore-size filters), treated with DNases (to remove extracellular DNA) and filtered onto 0.02 µm pore size filters. Finally, the filters were stained using SYBR Gold and analysed by epifluorescence microscopy^21^.

### Tangential Flow Filtration vs. vacuum filtration to concentrate viruses

For concentrating viral particles, supernatants obtained using the PC procedure (previously filtered through 0.2 μm pore size filters to remove cellular organisms; Whatman Anopore) were divided into two equal aliquots. One aliquot was filtered using 0.02 μm pore size filters (Anodisc 47 mm, Whatman) under vacuum, and the other one using a tangential flow filtration apparatus (Millipore Labscale TFF apparatus with a 100 KDa cartridge).

To minimize as much as possible the retention of viruses onto 0.2 µm pore size filters (Whatman Anopore) due to residual suspended mineral particles and cellular organisms, which may cause the clogging of the pores during filtration, filters were frequently changed during this step (typically we used 5–10 filters).

The efficiency of vacuum filtration *vs*. tangential flow filtration to concentrate viral particles was assessed by comparing the abundances of viruses recovered onto 0.02 μm pore size filters with those obtained from an aliquot of the retentate (final volume ca. 35 ml) resulting from the TFF.

### Extraction of viral DNA based on traditional vs. commercial kit protocols

Viruses extracted by using the PC procedure were concentrated onto 0.02 μm pore size filters after a pre-filtration step at 0.2 μm (see above). Filters containing the collected viruses were subjected to DNA extraction by using different procedures based on previously published laboratory protocols (the procedure reported by Thurber and the Sambrook’s method)^19, 33^ and a commercial kit (QIAamp DNA Micro Kit, QIAGEN; see details in the Supplementary Information). On the basis of the final DNA yield we selected the Sambrook’s method for the extraction and purification of viral DNA.

### Comparison between viromes generated with the PC procedure and the WB procedure

To assess whether the procedure used for the recovery of viruses could affect the viral assemblage composition, we carried out comparative analyses by using the PC and WB extraction procedures (as detailed above) on sediment samples collected in the same deep-sea site of the Mediterranean Sea.

### Viral assemblage composition of MDA treated and un-treated samples

Independent from the procedure used for viral recovery, the amounts of viral DNA isolated from the different benthic deep-sea sites with the exception of the Black Sea were not sufficient to be directly sequenced. Therefore, for a proper comparison all samples were amplified by Multiple Displacement Amplification (MDA) to increase the concentration of viral DNA suitable for metagenomic analysis^51^. Samples were processed with GE Healthcare GenomiPhi V2 kit (according to the manufacture instructions). After MDA, viral DNA was purified (using Wizard PCR and Gel Clean-up kit, Promega) and then sequenced onto a 454 Titanium FLX platform. To assess the potential biases induced by MDA, we compared MDA treated and un-treated viromes generated from sediment samples collected in the same deep-sea site of the Mediterranean Sea. To do so, DNA of viruses extracted by the PC procedure applied on 500 g of sediment was split into two aliquots, one of which amplified by MDA and the other was left untreated. Both DNA pools were then sequenced as reported above.

### Bioinformatic analyses

Taxonomic annotation of sequences at the domain level was carried out on MG-RAST^52^ using default parameters (10^−5^ of E-value cutoff, 60% identity percentage and 15 bp of alignment length on the RefSeq database for taxonomy and Subsystem database for putative functions)^52^.

Viral metagenomes were annotated at deeper taxonomic levels using the tool GAAS in MetaVir^16, 53^ by comparing reads against the RefSeq database of viral genomes with a threshold of 10^−5^ E-value. Rarefaction curves (at a clustering percentage of 75%) were also generated by using MetaVir both on all sequences and on a random subsample of 50,000 sequences.

The contribution of identified sequences and viral genotypes to each viral family (as number of either identified sequences or genotypes for each family to the total number of identified sequences and genotypes) was calculated for each virome. Viral families contributing for less than 0.1% to the assemblage in terms of sequences were not taken into account and the resulting number of viral genotypes in each family was used to represent the viral assemblage. Viral genotype richness was determined by counting the number of viral genotypes identified by MetaVir in each sample^54^. The contribution of different putative functions identified by the MG-RAST pipeline to each functional class was also determined in the same way.

Virome clustering was performed both by using tetranucleotide frequencies on the MetaVir server^16^ and by using the PRIMER-E 6 software with a distance matrix calculated using the Bray-Curtis similarity on the contribution of identified viral genotypes to viral families and on the contribution of identified functions to functional classes. A distance-based redundancy analysis (dbRDA) was performed to assess the relationships between the contribution of identified viral genotypes to viral families of the different viromes and environmental characteristics of the study sites.

The Nonpareil software^55^ was used to compute a diversity index (the Nonpareil index, which has been found to be correlated to other diversity indices used in metagenomics studies) not based on any reference database, to obtain a measure of the potential diversity of the whole dataset, which included both annotated and non-annotated sequences.

### Sequence quality check and assessment of the potential contamination from eukaryotic and prokaryotic DNA

The possible contamination by prokaryotic and eukaryotic cells and their DNA in the viral DNA pools was assessed by means of multiple approaches before and after sequencing. During the procedure of virus recovery, the presence of prokaryotic or eukaryotic cells was assessed by epifluorescence microscopy as reported above. After viral DNA extraction, the presence of prokaryotic and eukaryotic DNA was evaluated by quantitative PCR (qPCR) amplification using the TaqMan technology^56^ targeting prokaryotic 16 S rRNA and eukaryotic 18 S rRNA genes (see details in the Supplementary Information).

Raw sequences were first processed using PRINSEQ.^57^ to remove ambiguous residues and sequences with entropy scores <70 and to trim or remove sequences with a mean quality score <20; subsequently, they underwent de-replication with the UCLUST service provided by the MG-RAST v3 server^52^ to eliminate sequencing artifacts.

After these steps, all viromes generated in the present study were further checked for potential contamination by prokaryotic or eukaryotic DNA. The DeconSeq web server was used to remove non-viral sequences from high-quality viromes^58^, using a coverage to non-viral genomes >60% and an identity >80%. These thresholds were used to exclude as much as possible non-viral sequences, thus making the results more reliable. After contamination removal, the remaining virome sequences were further analysed by checking for the potential presence of bacterial, archaeal and eukaryotic rRNA genes with the hmm_rRNA detection function on the WebMGA server^59^.

A final comparison of dinucleotide frequencies of each clean virome was carried out with the PRINSEQ web server to compare dinucleotide frequencies of the viromes generated in the present study with those of previously published viromes and microbial metagenomes (which are pre-calculated and included in the program), to ensure that viromes generated in this study showed no prokaryotic genomic signatures.

### Data accessibility

High quality viromes can be accessed through the MetaVir server (metavir-meb.univ-bpclermont.fr/index.php?page=Welcome) under the project “EXPLODIVE”.

## Electronic supplementary material

Supplementary Information
