# Supplementary material for: From virus isolation to metagenome generation for investigating viral diversity in deep-sea sediments
Source: Sci Rep. 2017 Aug 21;7:8355. doi: 10.1038/s41598-017-08783-4 (PMC5566222; doi:10.1038/s41598-017-08783-4)
Supplement: Supplementary file 1 — Supplementary Information [file 41598_2017_8783_MOESM1_ESM.doc]

**Supplementary Information**

**From virus isolation to metagenome generation for investigating viral diversity in deep-sea sediments**

Cinzia Corinaldesi^1^*, Michael Tangherlini^2^, Antonio Dell’Anno^2^

^1^Department of Sciences and Engineering of Materials, Environment and Urbanistics Polytechnic University of Marche, Via Brecce Bianche 60131 Ancona Italy

^2^Department of Environmental and Life Sciences, Polytechnic University of Marche, Via Brecce Bianche, 60131 Ancona, Italy

**Supplementary Materials and Methods**

**Supplementary Table 1**

**Supplementary Figures 1-6**

**Supplementary references**

**Supplementary Materials and Methods**

*Sampling area and environmental characteristics*

Samples were collected during different oceanographic cruises conducted in the Black Sea in September 2006 (R/V Mare Nigrum), in the NE Atlantic 1 and NE Atlantic 2 sites in July-August 2005 and October 2008, respectively (R/V Discovery and Pelagia), in the Arctic Ocean in July-August 2005 (R/V Polarstern) and in the Mediterranean sea in April 2002 (R/V Urania).

After retrieval, sediment samples of the top 1 cm were collected using a sterile spatula and stored at -80°C until laboratory analyses (within 3 months). Anoxic sediment samples collected in the Black Sea were treated (and subsequently analyzed) under strictly anaerobic conditions (N_2_ atmosphere).

Temperature and salinity were measured in the different sites at the interface water-sediment by using with a SBE 3/F thermometer with 10^-3^ °Celsius resolution; and conductivity measurements were performed with a SBE-4 sensor.

The biopolymeric C content (used as a proxy of the more bioavailable fraction of organic C for heterotrophic consumers)^1^ was obtained by converting the carbohydrate, protein and lipid concentrations into carbon equivalents (using the conversion factors of 0.40, 0.49 and 0.75 μg C μg^-1^, respectively)^2^.

Proteins were extracted from sediments with NaOH (0.5 M, 4 h) and, after centrifugation (800 × g), the supernatant was analyzed spectrophotometrically according to Hartree (1972)^3^ modified by Rice (1982)^4^ to compensate for phenol interference. Protein concentrations were calculated from calibration curves of bovine serum albumin (ranging from 10 to 200 µg mL^-1^).

Carbohydrates were determined spectrophotometrically^5^ according to Gerchacov and Hatcher (1972)^6^. Carbohydrate concentrations were calculated from calibration curves of D-glucose (from 10 to 200 µg mL^-1^).

Lipids were extracted by direct elution with chloroform and methanol (1:1 V:V) and the resulting fraction, after evaporation in a dry hot bath at 80-100 °C for 20 min, was quantified according to the sulfuric acid-carbonization procedure^7^. Lipid concentrations were calculated from calibration curves of tripalmitine (from 10 to 100 µg mL^-1^). For each biochemical analysis, blanks were made with the same sediment samples previously treated in a muffle furnace (450°C, 4 h). All analyses were carried out in 3-5 replicates, using about 1 g of wet sediment.

Sampling sites were characterized by temperatures ranging from -0.51 to ca. 13°C (in Arctic and Mediterranean Sea sites, respectively) and salinity ranging from 22.1 to ca. 38.45 (in Black Sea and Mediterranean Sea sites).

All deep-sea sediment samples were largely dominated by the silt-clay fraction.

Sediments of NE Atlantic sites were characterized by a biopolymeric C content ranging from 2.3 to 5.7 mg g^-1^ of dry sediment, while those of the Arctic Ocean showed a biopolymeric C concentration of 5.9 mgC g^-1^ of dry sediment. Sediments of the Mediterranean site were characterized by a biopolymeric C concentration of 2.0 mgC g^-1^ of dry sediment. The Black Sea site, being a very peculiar deep-sea site due to anoxic and eutrophic conditions, was characterized by a much higher organic C content (104 mgC g^-1^ of dry sediment).

*Extraction of viral DNA based on traditional vs commercial kit protocols*

Different protocols were compared for the extraction of DNA from viruses once recovered by using the PC procedure. A protocol was based on physical-chemical lysis of viral capsids followed by DNA purification by phenol:chloroform and precipitation using isopropanol according to the Sambrook’s procedure^8^. Briefly, for viral DNA extraction each filter was added with a lysis buffer containing 20 mM EDTA, 10% SDS and 50 µg ml-1 proteinase K and then incubated at 56°C for 1 h. Another protocol was based on the use of a commercial kit (QIAmp DNA Micro Kit, QIAGEN), which is commonly used for the extraction and purification of DNA from small amounts of sample, according to the manufacture instructions. Additionally, we compared, using selected deep-sea sediment samples from the Black Sea and Arctic sites, the extraction efficiency of viral DNA obtained through the Sambrook’s method and the procedure reported by Thurber^9^, which combines different extraction reagents (formamide, SDS and proteinase K).

DNA concentrations were measured using a NanoDrop 3300 fluorometer and SYBR Gold as fluorochrome, and the DNA yields from the different protocols were compared. To evaluate the recovery efficiency of viral DNA of the extraction protocols in comparison with the expected amount of viral DNA (determined on the basis of the viral abundances measured by epifluorescence microscopy), viral abundances were converted into DNA concentration using an assumed mean DNA content per virus of 0.039 fg^10^.

*Quantitative PCR analyses*

The prokaryotic 16S rDNA sequences were amplified using the universal primers Uni340F (5′-CCTACGGGRBGCASCAG-3′) and Uni806R (5′-GGACTACNNGGGTATCTAAT-3′). The TaqMan probe was Uni516F (5′-TGYCAGCMGCCGCGGTAAHACVNRS-3′), which contained a fluorescent reporter dye (6-carboxyfluorescein) covalently attached to the 5′-end and a fluorescent quencher dye (6-carboxytetramethylrhodamine) attached six or more bases downstream of the reporter dye. The number in each primer or probe designation indicates the position of the 5′ end of the primer or probe in *Escherichia coli* 16S rRNA. To amplify prokaryotic genes, 40 PCR cycles were used: as 95 °C for 15 s, and 57 °C for 5 min, which were preceded by 3 min of Taq activation at 95 °C.

The eukaryotic 18S rDNA sequences were amplified using an equimolar mixture of two forward primers and two reverse primers (final concentration, 0.2 μM) and a TaqMan probe (final concentration, 0.1 μM), which were obtained from consensus alignment of eukaryotic sequences downloaded from the NCBI database. In particular, we used Euk f1 (5′-CGC AAG GCT GAA ACT TAA AG-3′), Euk f2 (5′-GTT GCA AAG CTG AAA CTT AAA G-3′), Euk r1 (5′-ATC ACT CCA CCA ACT AAG AAC-3′) and Euk r2 (5′-ATC GCT CCA CCA ACT AAG AA-3′). The forward primers correspond to the position 1119–1140 in the *Saccharomyces cerevisiae* (GenBank accession number Z75578), whereas the reverse primers correspond to the position 1281–1301. The TaqMan probe (Euk Probe; 5′-AAt TGA cGG AaG GgC-3′) corresponds to the position 1142–1156 in the *Saccharomyces cerevisiae*. This probe contained LNA (Locked Nucleic Acids represented by lower cases in the probe sequence) and was labelled with a fluorescent reporter dye (6-carboxyfluorescein) at the 5′end and a fluorescent quencher dye (BHQ1) at the 3′end. All of the qPCR was performed in a volume of 25 μL with an iQ5-icycler (Bio-Rad) using iQ Supermix (2×; Bio-Rad) containing 40 mm Tris-HCl, pH 8.4, 100 mm KCl, 0.4 mm each dNTP (dATP, dCTP, dGTP, dTTP), 50 U mL^−1^ hot-start iTaq DNA polymerase and 6 mm MgCl_2_. The thermal protocol used to amplify the eukaryotic genes was the same used for prokaryotic genes, except for the annealing temperature (60 °C for 1 min).

To quantify the 16S rDNA and 18S rDNA, calibration curves were obtained from a standard solution of *E. coli* and Herring testes DNA, respectively (from 0.2 to 200 pg µL^-1^). Standard concentrations were plotted against the number of cycles at which the fluorescence signal increased above background, or the cycle threshold (the Ct value). The iCycler software analysis programme was used to calculate the Ct values and to determine the sample concentrations based on the standard curves. All samples, standards, and negative controls were analyzed in triplicate qPCR reactions.

Since no contamination was detected in the samples investigated, considering a detection limit of the qPCR experimentally determined of 25-30 ribosomal gene copies, the procedure utilized here allowed us to recover exclusively viral DNA suitable for pyrosequencing analyses (by 454 Titanium FLX platform) or possibly for other sequencing platforms.

**Table S1.** Table containing the number of reads pre-quality (Pre-QC), post-quality (Post-QC) check, after de-replication and after contamination removal by DeconSeq, sequences with a match to known viral genomes, mean length of the reads, mean GC content, and total number of sequenced bases obtained by pyrosequencing of viral metagenomes from the benthic deep-sea systems investigated.

|  | **Read abundance** | | | | |  |  |  |
| --- | --- | --- | --- | --- | --- | --- | --- | --- |
| **Sampling sites** | **Pre-QC** | **Post-QC** | **After de-replication** | **After contamination removal** | **Viral sequences** | **Read mean length (bp)** | **Mean GC content (%)** | **Sequenced bases (bp)** |
| ***Black Sea*** | *205938* | *172954* | *78436* | *78436* | *14847* | *486* | *42* | *38194503* |
| ***NE Atlantic 1*** | *169762* | *142852* | *107102* | *107090* | *3588* | *524* | *49* | *56186871* |
| ***NE Atlantic 2*** | *224193* | *194670* | *165518* | *165517* | *13906* | *484* | *45* | *80252739* |
| ***Arctic*** | *147478* | *103084* | *79666* | *79646* | *5221* | *484* | *44* | *38593122* |
| ***Mediterranean Sea*** | *250619* | *218222* | *76219* | *65340* | *9310* | *542* | *48* | *35443711* |

**Figure S1.** Comparison of dinucleotide frequencies between deep-sea viromes generated in the present study (red circles) and other published viromes (blue circles, on the right) and microbial metagenomes (blue circles, on the left).


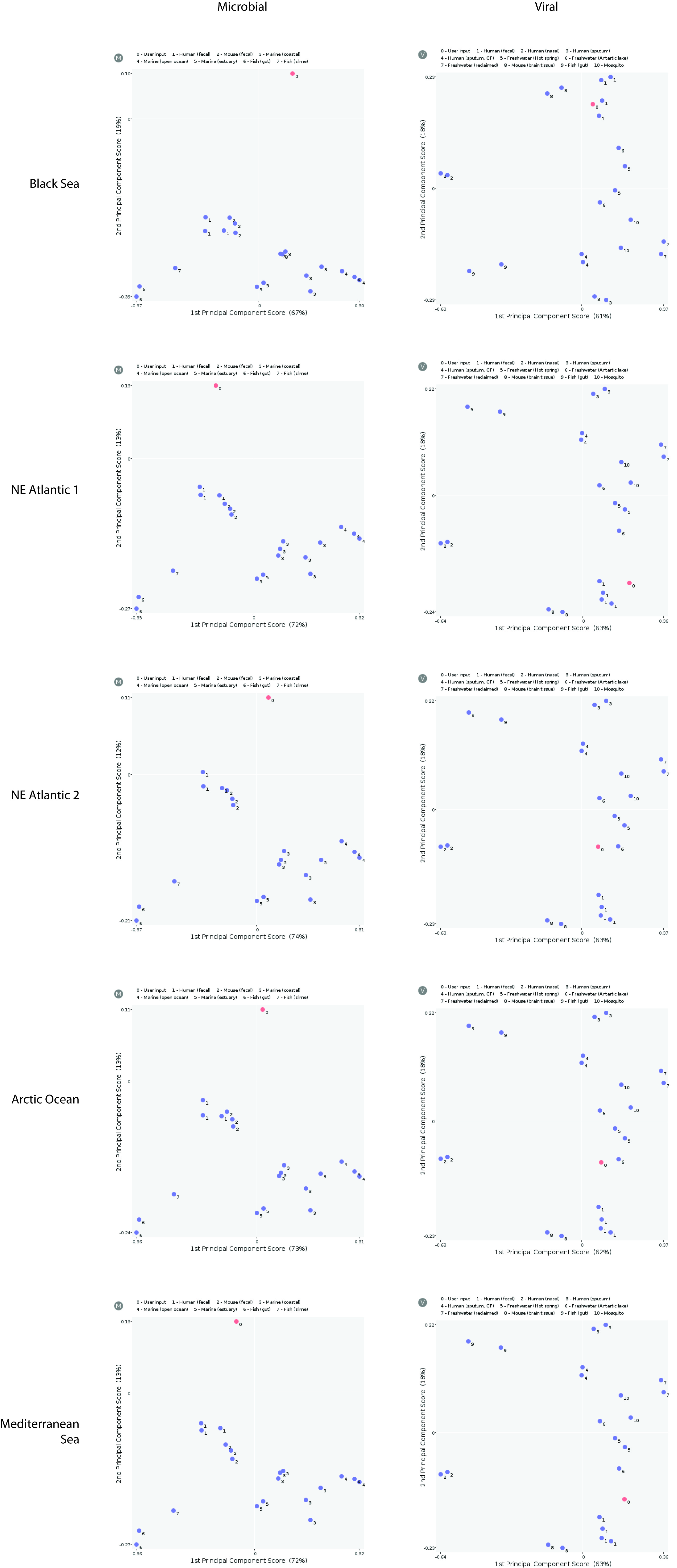


**Figure S2.** Nonpareil diversity indices calculated for each deep-sea sediment virome.


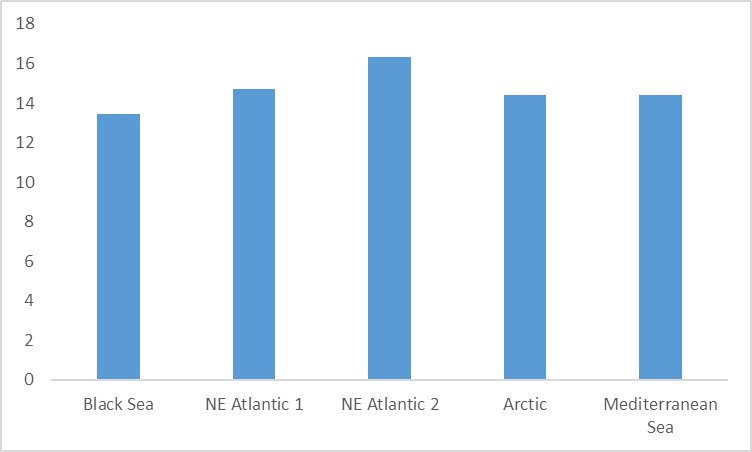


**Figure S3.** Cluster analysis of the viral taxonomic composition in terms of contribution of different viral genotypes to each viral family obtained by using the Bray-Curtis similarity (A), and cluster analysis based on the tetranucleotide frequencies determined by MetaVir server (B) .


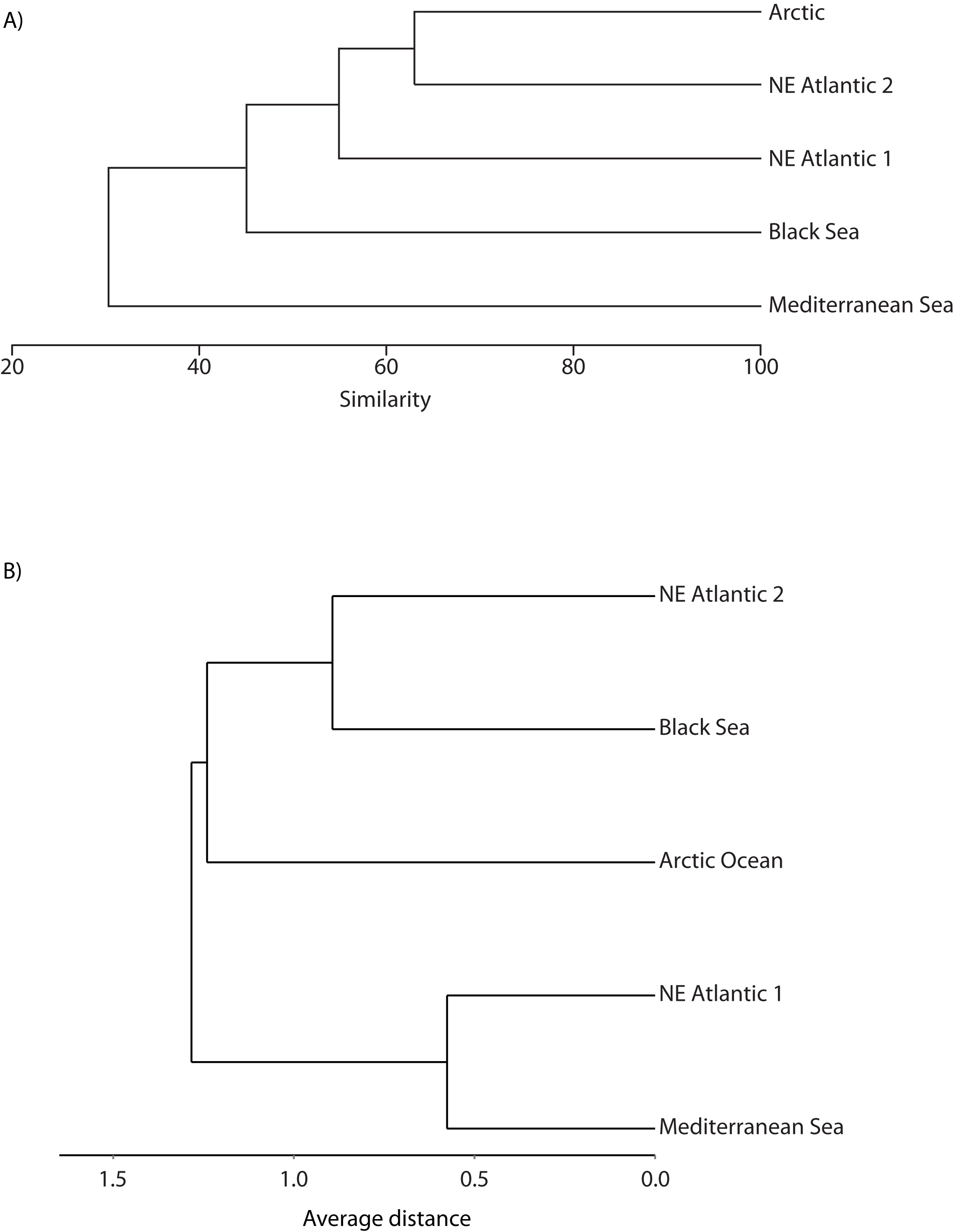


**Figure S4.** Output of the distance-based redundancy analysis (dbRDA) carried out to investigate the relationships between environmental parameters and viral assemblage compositions of the different benthic ecosystems.

**
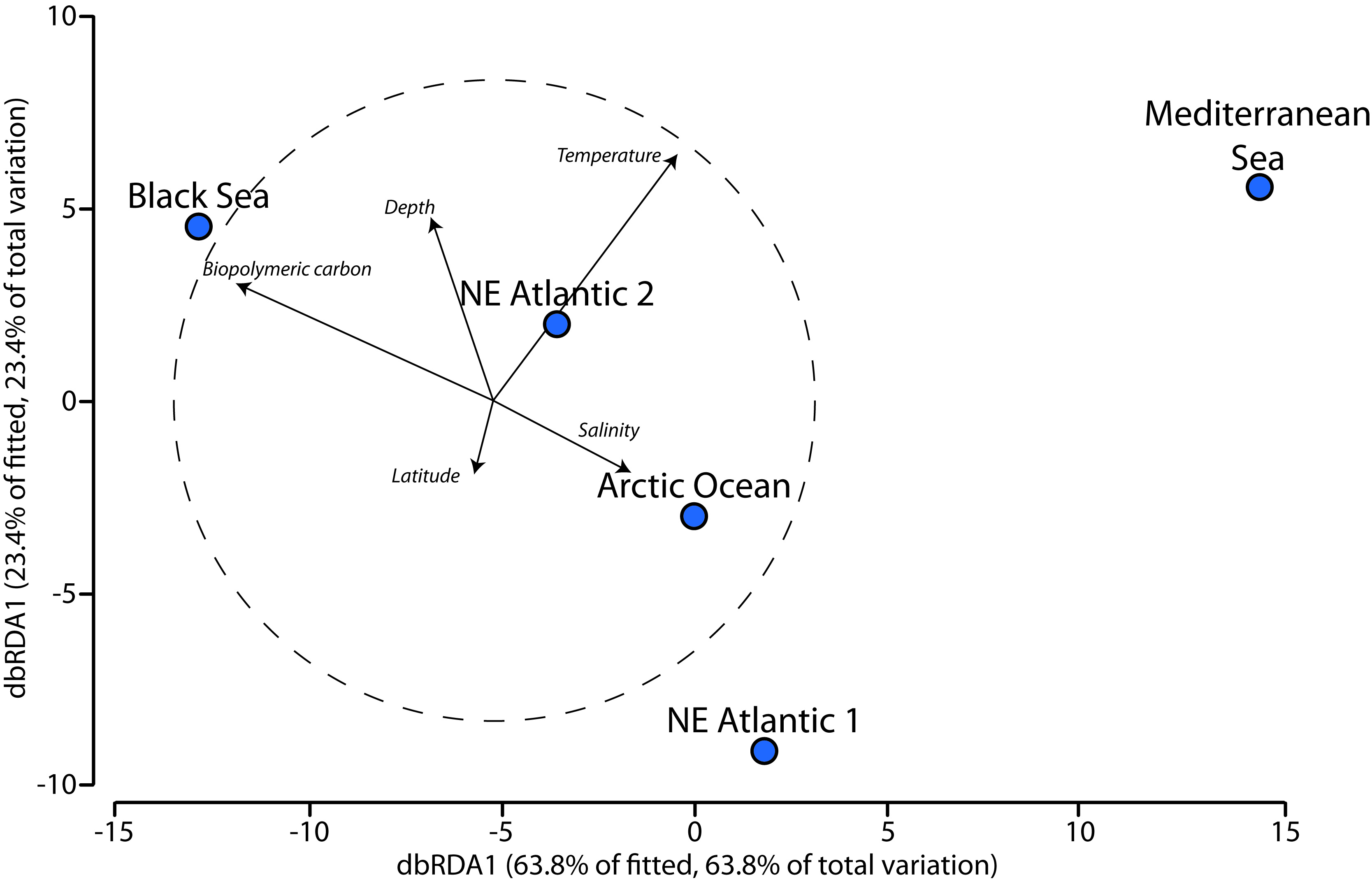
**

**Figure S5.** Rarefaction curve analysis carried out on the five viromes generated in the present study. A) Curves calculated on all the metagenomics sequences. B) Curves calculated on a randomly subsampled fraction of 50000 sequences.

**
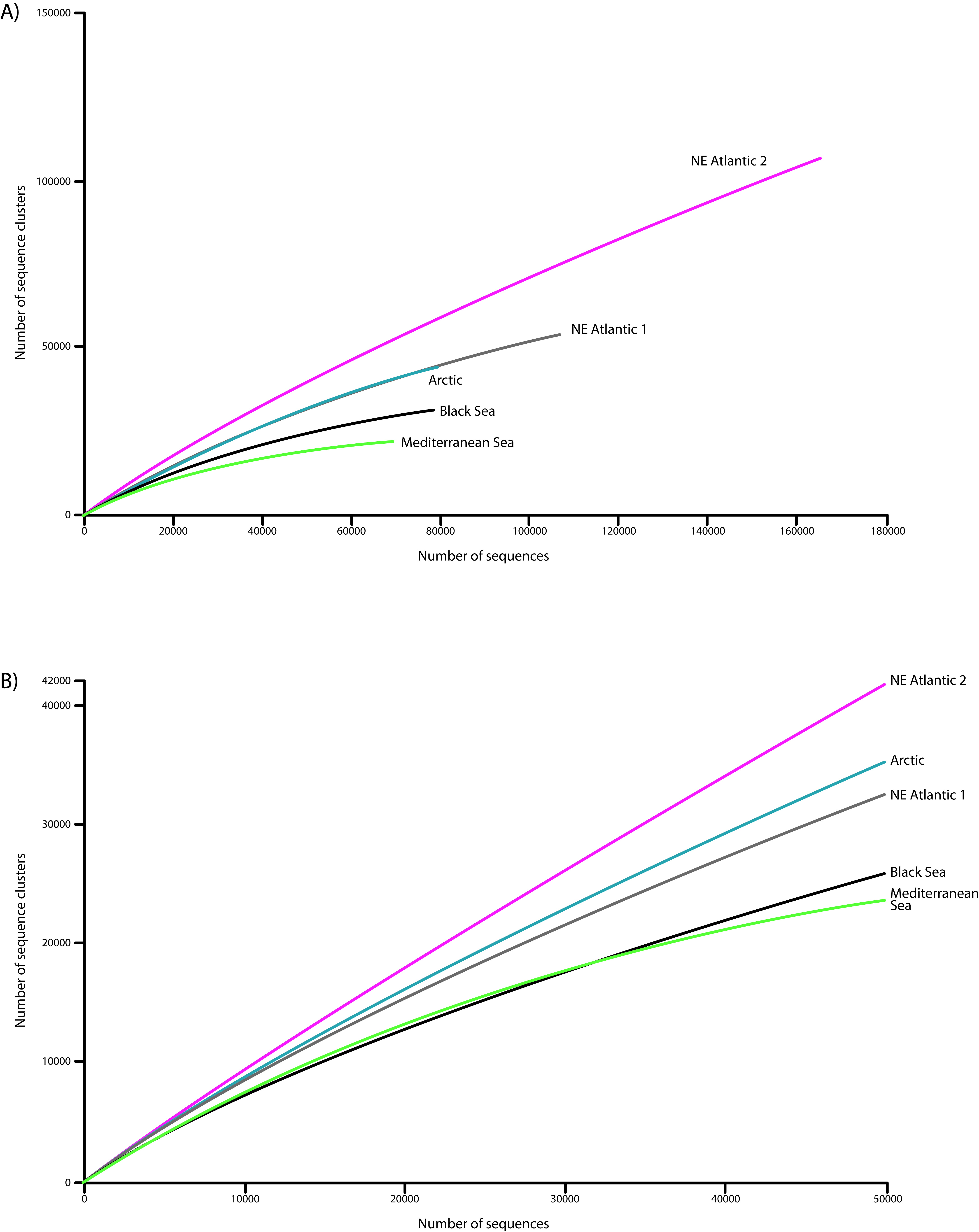
**

**Figure S6.** Cluster analyses based on the Bray-Curtis similarity of the viral putative functions (expressed as contribution of putative viral functions to the different functional classes).


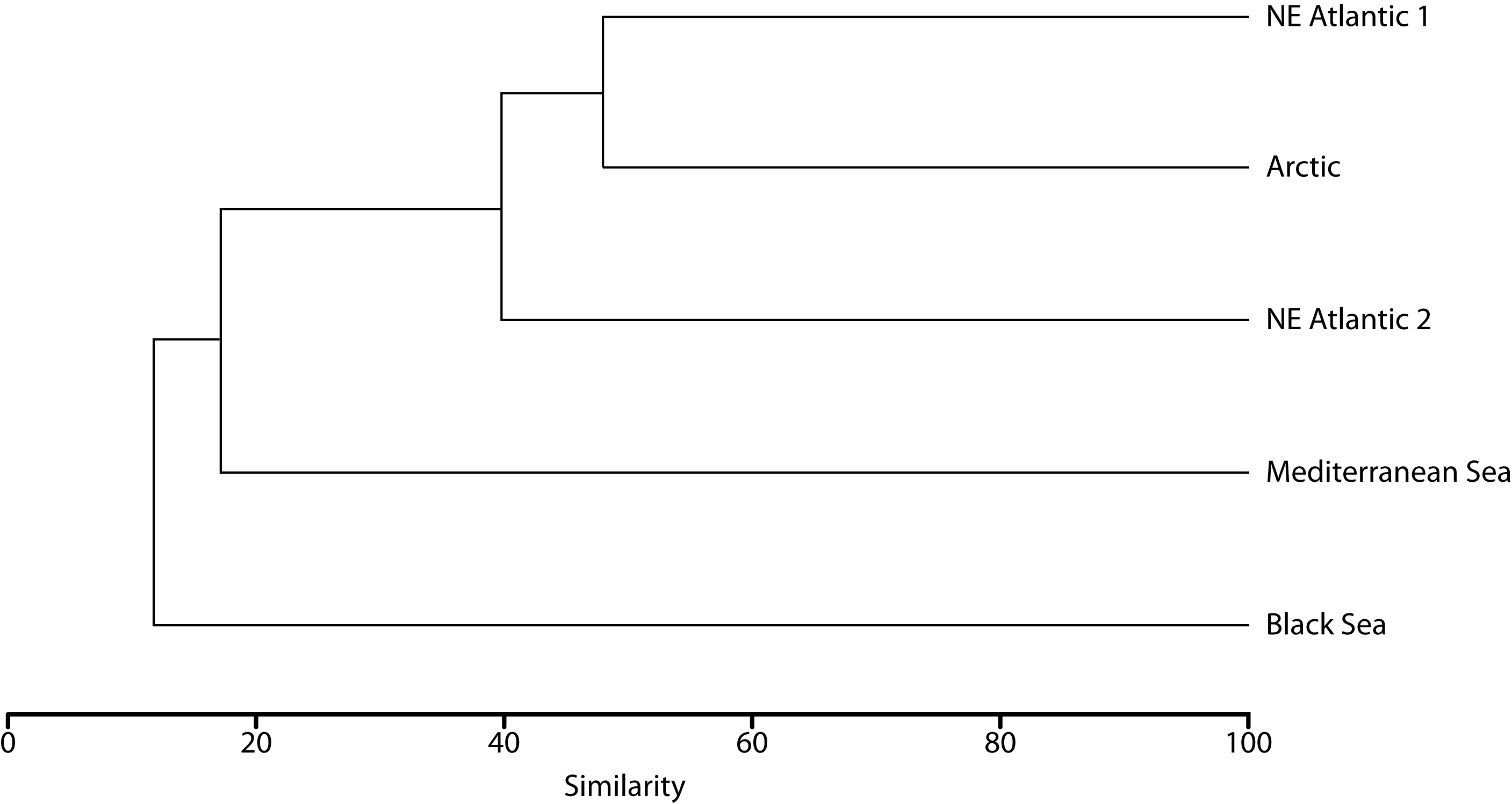


**Supplementary references**

1. Pusceddu, A., Dell’Anno, A., Fabiano, M., & Danovaro, R. (2009) Quantity and bioavailability of sediment organic matter as signatures of benthic trophic status. *Marine Ecology Progress Series*, 375, 41-52.
2. Danovaro, R. in *Methods for the study of deep-sea sediments, their functioning and biodiversity* (CRC Press, 2010)
3. Hartree, E. F. (1972) Determination of protein: a modification of the Lowry method that gives a linear photometric response. *Analytical Biochemistry*, 48, 422-7.
4. Rice, D. L. (1982) The detritus nitrogen problem: new observations and perspectives from organic geochemistry. *Marine Ecology Progress Series,* 9, 153-162.
5. Dubois, M., Gilles, K. A., Hamilton, J. K., Rebers, P., & Smith, F. (1956) Colorimetric method for determination of sugars and related substances. *Analytical Chemistry*, 28, 350-356.
6. Gerchacov, S. M., & Hatcher, P. G. (1972) Improved technique for analysis of carbohydrates in sediments. *Limnology and Oceanography*, 17, 938–943
7. Marsh, J. B., & Weinstein, D. B. (1966) Simple charring method for determination of lipids. Journal of Lipid Research, 7, 574-576.
8. Sambrook, J., E. F. Fritsch, and T. Maniatis. in *Molecular cloning*, 14-19 (Cold spring harbor laboratory press, 1989).
9. Thurber, R. V., Haynes, M., Breitbart, M., Wegley, L., & Rohwer, F. Laboratory procedures to generate viral metagenomes. *Nat Protoc* **4**, 470-483 (2009).
10. Paul, J. H., & Sullivan, M. B. Marine phage genomics: what have we learned? *Curr* *Opin Biotechnol* **16**, 299-307 (2005).
